# A randomized controlled trial of vitamin E and selenium on rate of decline in lung function

**DOI:** 10.1186/s12931-015-0195-5

**Published:** 2015-03-11

**Authors:** Patricia A Cassano, Kristin A Guertin, Alan R Kristal, Kathryn E Ritchie, Monica L Bertoia, Kathryn B Arnold, John J Crowley, JoAnn Hartline, Phyllis J Goodman, Catherine M Tangen, Lori M Minasian, Scott M Lippman, Eric Klein

**Affiliations:** Division of Nutritional Sciences, Cornell University, 209 Savage Hall, Ithaca, NY 14853 USA; Department of Healthcare Policy and Research, Division of Biostatistics and Epidemiology, Weill Cornell Medical College, New York, NY USA; Current address: Nutritional Epidemiology Branch, Division of Cancer Epidemiology and Genetics, National Cancer Institute, NIH, Department of Health and Human Services, Bethesda, MD USA; Department of Epidemiology, University of Washington, Seattle, WA USA; Fred Hutchinson Cancer Research Center, Seattle, WA USA; Current address: Loyola Department of Oral and Maxillofacial Surgery, Loyola University, Chicago, IL USA; Current address: Department of Nutrition, Harvard T.H. Chan School of Public Health, Harvard University, Boston, MA USA; SWOG Statistical Center, Seattle, WA USA; Division of Cancer Prevention, National Cancer Institute, Bethesda, MD USA; University of California San Diego, Moores Cancer Center, La Jolla, CA USA; Cleveland Clinic, Cleveland, OH USA

**Keywords:** Spirometry, Vitamin E, Selenium, Forced expiratory volume, Forced expiratory flow rate

## Abstract

**Background:**

The intake of nutrients with antioxidant properties is hypothesized to augment antioxidant defenses, decrease oxidant damage to tissues, and attenuate age-related rate of decline in lung function. The objective was to determine whether long-term intervention with selenium and/or vitamin E supplements attenuates the annual rate of decline in lung function, particularly in cigarette smokers.

**Methods:**

The Respiratory Ancillary Study (RAS) tested the single and joint effects of selenium (200 μg/d *L*-selenomethionine) and vitamin E (400 IU/day *all rac*-α-tocopheryl acetate) in a randomized double-blind placebo-controlled trial. At the end of the intervention, 1,641 men had repeated pulmonary function tests separated by an average of 3 years. Linear mixed-effects regression models estimated the effect of intervention on annual rate of decline in lung function.

**Results:**

Compared to placebo, intervention had no main effect on either forced expiratory volume in the first second (FEV_1_) or forced expiratory flow (FEF_25–75_). There was no evidence for a smoking by treatment interaction for FEV_1_, but selenium attenuated rate of decline in FEF_25–75_ in current smokers (*P* = 0.0219). For current smokers randomized to selenium, annual rate of decline in FEF_25–75_ was similar to the annual decline experienced by never smokers randomized to placebo, with consistent effects for selenium alone and combined with vitamin E.

**Conclusions:**

Among all men, there was no effect of selenium and/or vitamin E supplementation on rate of lung function decline. However, current smokers randomized to selenium had an attenuated rate of decline in FEF_25–75_, a marker of airflow.

**Trial registration:**

Clinicaltrials.gov identifier: NCT00241865.

**Electronic supplementary material:**

The online version of this article (doi:10.1186/s12931-015-0195-5) contains supplementary material, which is available to authorized users.

## Background

Pulmonary function, which is reliably measured by spirometry, is central to the diagnosis and staging of chronic obstructive pulmonary disease (COPD). COPD, the third most common cause of death in the US, led to $29.5B in direct costs and $20B in lost productivity costs in 2010 [1]. The age-related rate of decline in the forced expiratory volume in the first second (FEV_1_) is a marker of mortality risk in the general population [2] and in healthy smokers [3], the rate of decline is steeper in smokers [4] and in COPD patients, although the latter association varies by disease attributes [5,6]. The forced expiratory flow at the mid-portion of forced vital capacity (FEF_25–75_) reflects the state of small airways, and offers a measure of lung function reflecting airflow rather than volume [7]. Attenuating lung function decline may reduce morbidity and mortality, both in healthy persons and in COPD patients. The identification of factors that affect lung function decline is important to the development of clinical or public health interventions.

Both smoking and aging accelerate the annual rate of decline in FEV_1_ [4,8]; the effect of other factors, including diet, is less clear [9]. Observational studies of nutrients (whole foods, micronutrients, dietary patterns, biomarkers) and lung outcomes (COPD, lung function) support the broad hypothesis that nutrients with antioxidant properties improve lung health [10-13], presumably by altering the oxidant/antioxidant balance in lung tissue. A recent critical review of the evidence for a causal relation between nutrition and lung outcomes [9] concluded there was a “limited/suggestive” role for diet, reflecting that the majority of studies are cross-sectional. However, most existing longitudinal studies report protective associations of antioxidant nutrients and lung outcomes, and there is strong evidence that the beneficial effects of diet may be limited to smokers [14]. Given that observational studies are limited by potential confounding due to lifestyle factors associated with healthful diets, and given that measures of diet based on self-report are subject to bias and have poor precision, experimental studies are needed to fully understand whether nutrition affects lung function and its decline with age, particularly in cigarette smokers.

The few randomized controlled trials considering respiratory endpoints other than lung cancer used post-hoc, secondary analyses [15-17] mainly in special populations (all heavy smokers, all with vascular disease), did not study lung function decline, and did not test selenium. Using the infrastructure of the Selenium and Vitamin E Cancer Prevention Trial (SELECT) [18], we conducted an ancillary study nested within SELECT to investigate the *a priori* hypothesis that supplementation with selenium and/or vitamin E, two nutrients with antioxidant potential, would attenuate the annual decline in lung function; we hypothesized a stronger effect in cigarette smokers given their higher exposure to inhaled oxidants.

## Methods

### Study design

The Respiratory Ancillary Study (RAS) was nested within SELECT [18], a phase 3 randomized, placebo-controlled double-blind trial of 35,533 men testing whether selenium (200 μg/d *L*-selenomethionine) and vitamin E (400 IU/d *all rac-*α-tocopheryl acetate) alone and/or in combination would prevent prostate cancer. SELECT eligibility included age ≥55 y (≥50 y in African-Americans), serum prostate-specific antigen ≤ 4 ng/mL, and no clinical evidence of prostate cancer. SELECT enrolled men in the United States and Canada between 2001–2004; use of study supplements stopped on 10/23/2008, after an interim analysis determined that there was no effect and that further intervention was unlikely to show significant reduction in prostate cancer incidence [18]. RAS used a post-randomization design, due to rapid enrollment in SELECT relative to the start of RAS; thus, we did not measure pre-randomization lung function, but we captured the rate of decline over the intervention period through repeated measurements of lung function. This design assumed that the intervention effect is reached early in the study, and is stable over time. To test the hypothesis that current smokers benefit more from intervention, RAS enrolled men from the 16 SELECT sites with the greatest number of current cigarette smokers (Additional file 1: Table S1). Based on the predicted effect of intervention on annual FEV_1_ decline, assuming a 7–10 year follow-up, the target sample size in RAS was 3000 men.

### Participants

Eligibility requirements for registration to RAS included SELECT registration at one of the 16 SELECT sites with a high proportion of smokers and adherence to supplements (either active or placebo) at the time of RAS registration. Each SELECT site invited all eligible current smokers to RAS and, depending on the number of participants at the site, either a random sample or all eligible former and never smokers. Ultimately, men were registered at their first (5%), second (17%), third (38%) or fourth (40%) annual SELECT visit. The RAS was approved by local IRBs at each of the 16 study sites, and by the Cornell University IRB.

### End point assessment

The primary endpoint was annual decline in FEV_1_; the secondary endpoint was annual decline in FEF_25–75_. Spirometry was assessed at three out of four annual visits spanning three years. Due to early termination of SELECT, not all RAS participants completed all scheduled pulmonary function tests (PFTs); the endpoint (the third and final PFT) was available for 57% of participants. We assume this is an unbiased sample given the timing of supplementation withdrawal relative to the timing of a participant’s annual visit is expected to be random and thus equal across arms.

Pulmonary function testing followed American Thoracic Society guidelines [19] and used the EasyOne handheld, flow-sensing spirometer, which has excellent validity and reliability, and significantly simpler field implementation in comparison to desktop devices [20]. Only PFTs meeting criteria for acceptable start and end of test and for reliability were included in analyses; further details are provided in Additional file 1: Table S2).

### Statistical analysis

There were three pre-specified main effect comparisons between each active treatment arm and placebo, with a *P*-value threshold of 0.018 to account for the three tests with a common placebo group. All analyses were intent-to-treat, and effects were estimated using a linear mixed-effects regression model incorporating the repeated measurements of pulmonary function (either FEV_1_ or FEF_25–75_) as the outcome. The model included random intercept and slope, and the following fixed effects: time (time between each PFT and baseline), treatment arm and its interaction with time (treatment-by-time), age, height, race and smoking. The treatment-by-time coefficient estimated the effect of treatment on annual rate of decline in lung function. All RAS men with ≥1 (n = 2920) PFT were included in the model and contributed to estimates of effects of age, height, race and smoking, but only men with ≥2 PFTs (n = 1677) were informative for the estimate of the time-by-treatment coefficient. Missing data were assumed to be missing at random, given very low drop-out rates. To test whether treatment effects differed by smoking status, models were extended to include the treatment-by-time-by-smoking interaction terms, and the significance threshold for the interaction effects was a nominal *P*-value of 0.05.

## Results

### Participants

**Figure 1:**
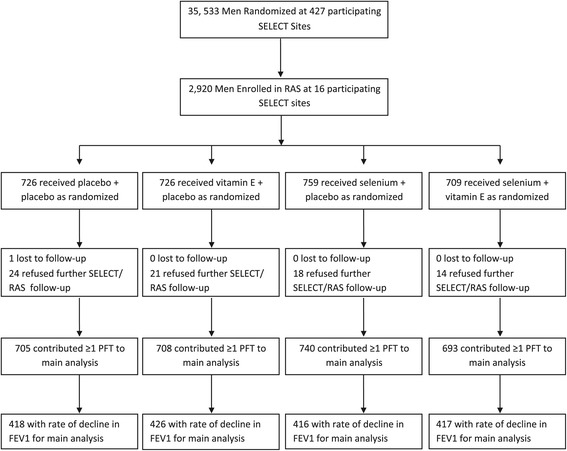
**Flow of participants included in FEV**_**1**_ **analysis by intervention group.** ^1^Participants lost to follow-up defined as deaths and participants not in contact for 2 years prior to the end of supplemented time period (March 1, 2009 was used in analyses to proxy the end of supplement). ^2^Refusals defined as withdrawals from SELECT and/or RAS.

RAS enrolled 2,920 men (Figure 1) at 16 SELECT sites between 7/2/2004 and 4/30/2007. RAS eligibility and enrollment were blind to intervention arm, and indeed the number of participants across the four arms was balanced (Figure 1). RAS experienced minimal attrition, with only 2-3% of men in each intervention group refusing further RAS and/or SELECT follow-up at some time point after registration. All participants studied herein had at least one acceptable PFT, and 56 to 60% of participants in each arm had repeated PFTs, confirming that repeated measurements for the endpoint assessment were similar by arm. The mean number of PFTs per participant was 2.3 (SD 1.1; median 3) with a mean of 36.1 months (SD 4.2) between first and last PFT (median 35.8 months; range 24 to 52). Spirometry quality control scores ranged from 3.2 to 3.7 out of maximum score of 4 (Additional file 1: Table S2).

RAS participants had similar distributions of age, race/ethnicity, education, smoking history and height across intervention arms (Table 1), confirming that the post-randomization design yielded four groups balanced on characteristics. The participants with one PFT were similar on all baseline characteristics to participants with repeated PFTs (data not shown); this is consistent with our expectation because the lack of repeated PFT data was a function of the date the intervention was withdrawn, and was not driven by participant choice or participant characteristics. Thus, we expect the estimate of the effect of treatment on lung function decline to be unbiased. Among participants who completed the final PFT, the mean time from SELECT registration to a participant’s last PFT was 60.4 months (SD 10.8; median 59.8), thus results reflect intervention effects of about 5 years duration.

**Table 1:** **Baseline characteristics of study participants** ^**†**^

**Characteristic:**	**Placebo (n = 726)**	**Vitamin E (n = 726)**	**Selenium (n = 759 )**	**Vitamin E + selenium (n = 709)**
**Age at SELECT randomization, years**				
Median (interquartile range)	61.7 (57.6–67.5)	61.5 (57.6–66.2)	61.6 (57.7–66.8)	61.5 (57.9–66.6)
50-54	56 (8)	48 (7)	54 (7)	48 (7)
55-64	418 (58)	459 (63)	466 (62)	435 (61)
65-74	212 (29)	190 (26)	208 (27)	192 (27)
≥75	40 (6)	29 (4)	31 (4)	34 (5)
**Race/ethnicity**				
White	510 (70)	491 (68)	515 (68)	489 (69)
African American	165 (23)	177 (24)	189 (25)	162 (23)
Hispanic (non-African American)	20 (3)	32 (4)	22 (3)	22 (3)
Hispanic (African American)	6 (1)	3 (<1)	5 (<1)	5 (1)
Other	25 (3)	23 (3)	28 (4)	31 (4)
**Education (highest level)**				
≤High school graduate or GED	157 (22)	172 (24)	176 (23)	169 (24)
Some college/vocational school	210 (29)	221 (30)	216 (28)	203 (29)
≥College graduate	348 (48)	327 (45)	361 (48)	335 (47)
Unknown/missing	11 (1)	6 (1)	6 (1)	2 (<1)
**Smoking status**				
Never	262 (36)	239 (33)	280 (37)	236 (33)
Current	113 (15)	123 (17)	108 (14)	114 (16)
Former	338 (47)	349 (48)	363 (48)	351 (50)
Ever (Unknown current status)^‡^	6 (1)	9 (1)	5 (1)	7 (1)
Unknown	7 (1)	6 (1)	3 (<1)	1 (<1)
**Height at SELECT Randomization** (cm), mean (standard deviation)	176.8 (8.2)	176.2 (7.8)	176.8 (7.3)	176.4 (7.8)

^†^Number (percent) unless otherwise noted.

^‡^Participants who were ever smokers, but did not report current status to differentiate current versus former.

Adherence among RAS participants (Table 2), determined using pill count, compares well to all SELECT participants [18]. Across the four arms, 87 to 92% of RAS men were adherent to the selenium supplement (or matching placebo) in year 1, and 80 to 84% were adherent in year 5. Similarly, 87 to 92% of RAS men were adherent to the vitamin E supplement (or matching placebo) in year 1, and 79 to 81% were adherent in year 5. Across all arms, for the full study period, self-supplementation with non-study vitamin E and selenium (drop-in rate, assessed by self-reported use of either supplement) was reported by ≤2.3% and ≤1.2% of participants, respectively.

**Table 2:** Adherence* to study supplements by pill counts

	**Placebo**	**Vitamin E**	**Selenium**	**Selenium + vitamin E**
**Selenium/matching placebo**		**% adherent (range)** ^**†**^		
Year 1 (n = 2,920)	92 (87–92)	89 (84–90)	91 (85–91)	87 (82–88)
Year 2 (n = 2,920)	90 (84–91)	87 (81–87)	89 (83–90)	87 (81–88)
Year 3 (n = 2,910)	90 (83–91)	87 (81–88)	89 (82–89)	87 (80–88)
Year 4 (n = 2,887)	85 (78–87)	84 (76–85)	86 (77–87)	87 (77–88)
Year 5 (n = 2,216)	84 (76–85)	80 (73–82)	82 (75–84)	83 (73–85)
Year 6 (n = 901)	82 (72–85)	79 (68–82)	82 (74–84)	86 (75–87)
**Vitamin E/matching placebo**		**% adherent (range)** ^**†**^		
Year 1 (n = 2,920)	92 (87–92)	89 (84–90)	90 (86–91)	87 (82–88)
Year 2 (n = 2,920)	90 (85–91)	88 (81–88)	88 (82–89)	87 (80–88)
Year 3 (n = 2,910)	88 (82–89)	87 (81–88)	86 (79–87)	86 (78–87)
Year 4 (n = 2,887)	84 (76–85)	81 (74–83)	84 (76–86)	84 (75–86)
Year 5 (n = 2,216)	81 (74–83)	79 (71–81)	81 (74–83)	79 (69–81)
Year 6 (n = 901)	81 (69–83)	76 (66–79)	78 (70–81)	80 (69–82)

*Adherence defined as report of taking ≥80% of study pills; decreasing denominators over time reflect varying amounts of follow-up on participants.

^†^Percent adherent (range); percent calculated for all participants with data, and ranges estimated by including participants with missing data and assuming missing were either all non-adherent (low estimate) or all adherent (high estimate).

### Rate of decline in pulmonary function

Overall, the distribution of rate of decline in FEV_1_ was consistent with expectations of decline, and the mean annual change in FEV_1_ was −37.5 mL (SD 12.5; Additional file 1: Figure S1). Compared to never smokers, FEV_1_ was 363 mL lower in current smokers and annual decline in FEV_1_ was 6.9 mL/y steeper. In unadjusted analyses of raw data, compared with the placebo group (Table 3), participants randomized to intervention experienced an attenuation of between 3 and 6 mL/y in rate of change in FEV_1_, but there were no statistically significant differences between arms. Similarly, for rate of decline in FEF_25–75_ (mL/second/y), there were no statistically significant differences between arms (Table 4).

**Table 3:** **Model-based estimated mean annual decline in FEV**_**1**_ **(mL/year) by treatment group in the full sample, and stratified by cigarette smoking status (Additional file** 1 **: Tables S3 and S4 for model coefficients)**

	**Full sample**			Stratified by smoking status*		
**All treatment groups:**	**Unadjusted**	**Adjusted (model-based)**	***P*** **value** ^**a**^	**Never smokers**	**Former smokers**	**Current smokers**
	**Mean (SD)** ^**†**^	**Mean (95% CI)** ^**††**^		**Mean (95% CI)** ^**††**^	**Mean (95% CI)** ^**††**^	**Mean (95% CI)** ^**††**^
Placebo	−41 (74)	−39 (−46, −32)	reference	−40 (−52, −29)	−35 (−45, −25)	−51 (−67, −35)
Vitamin E	−34 (75)	−33 (−40, −26)	0.1866	−31 (−42, −20)	−36 (−46, −26)	−29 (−44, −14)
Selenium	−37 (74)	−38 (−44, −31)	0.7502	−37 (−48, −26)	−38 (−48, −28)	−38 (−54, −23)
Vitamin E and Selenium	−34 (74)	−34 (−41, −28)	0.3133	−32 (−44, −20)	−32 (−41, −22)	−44 (−59, −29)
**Marginal Models** ^**b**^ **:**						
Any Selenium versus Placebo	Placebo	−39 (−46, −32)	reference	−40 (−52, −29)	−35 (−45, −25)	−49 (−66, −33)
	Any Selenium	−36 (−41, −31)	0.4442	−35 (−43, −27)	−35 (−42, −28)	−42 (−52, −31)
Any Vitamin E versus Placebo	Placebo	−39 (−42, −36)	reference	−40 (−51, −29)	−34 (−44, −24)	−49 (−66, −33)
	Any Vitamin E	−34 (−38, −30)	0.1772	−31 (−40, −23)	−34 (−41, −27)	−37 (−47, −26)

*Abbreviations*: *FEV*_*1*_ forced expiratory volume in the first second, *mL* milliliter.

*All smoking-stratified treatment vs. placebo comparisons exceeded the *P* value threshold of 0.05 (all values ≥ 0.25); in current smokers vitamin E vs. placebo P = 0.3552, selenium vs. placebo P = 0.4985, vitamin E + selenium vs. placebo P = 0.8887, any selenium vs. placebo P = 0.8705.

^†^Mean decline (SD); calculated for men with ≥2 years between first and last pulmonary function tests where date of last test ≤ March 2009; calculated in 418 (placebo), 426 (vitamin E), 416 (selenium) and 417 (vitamin E + selenium) participants.

^††^Model-based estimated mean decline and 95% confidence interval (Additional file 1: Tables S3 and S4 for regression model results); main effects models adjusted age, height, race, smoking status, time and tested time x treatment effect (effect of treatment on slope); smoking interaction models adjusted age, height, race, smoking status, and all two way interactions to test smoking x time x treatment interaction (effect of treatment on slope differs by smoking).

^a^P value from mixed-effects linear regression model, with placebo reference group.

^b^Marginal models combine treatment groups. Any selenium model compares participants on any selenium (selenium alone and in combination with E) to placebo. In a separate model, participants on any vitamin E (vitamin E alone and in combination with selenium) are compared to placebo.

**Table 4:** **Model-based estimated annual decline in FEF**_**25–75**_ **(mL/second/year) by treatment group in the full sample, and stratified by cigarette smoking status (Additional file** 1 **: Tables S3 and S4 for model coefficients)**

	**Full sample**			**Stratified by smoking status**		
**All treatment groups:**	**Unadjusted**	**Adjusted (model-based)**	***P*** **value** ^**a**^	**Never smokers**	**Former smokers**	**Current smokers**
	**Mean (SD)** ^**†**^	**Mean (95% CI)** ^**††**^		**Mean (95% CI)** ^**††**^	**Mean (95% CI)** ^**††**^	**Mean (95% CI)** ^**††**^
Placebo	−63 (191)	−61 (−78, −43)	reference	−44 (−73, −15)	−58 (−82, −32)	−106 (−148, −63)
Vitamin E	−45 (201)	−46 (−66, −29)	0.2739	−47 (−76, −18)	−42 (−67, −17)	−59 (−95, −21)
Selenium	−67 (200)	−64 (−81, −47)	0.6620	−67 (−95, −38)	−75 (−100, −49)	−45 (−85, −5)*
Vitamin E and Selenium	−45 (190)	−47 (−64, −29)	0.2719	−71 (−102, −40)	−30 (−55, −5)	−50 (−90, −10)*
**Marginal Models** ^**b**^ **:**						
Any Selenium versus Placebo	Placebo	−61 (−78, −43)	reference	−44 (−73, −15)	−57 (−82, −32)	−106 (−148, −63)
	Any Selenium	−56 (−69, −44)	0.7020	−68 (−90, −47)	−52 (−70, −34)	−47 (−76, −19)*
Any Vitamin E versus Placebo	Placebo	−61 (−78, −43)	reference	−44 (−73, −15)	−57 (−82, −32)	−105 (−147, −63)
	Any Vitamin E	−46 (−58, −34)	0.2058	−59 (−80, −38)	−36 (−54, −18)	−54 (−81, −27)*

*Abbreviations*: *FEF*_*25–75*_ forced expiratory flow rate from the 25^th^ to 75^th^ percentile of the forced vital capacity, *mL* milliliter.

*P < 0.05; more specifically, in *current smokers* selenium vs. placebo P = 0.0219, vitamin E + selenium vs. placebo P = 0.0236, any selenium vs. placebo P = 0.0095, any vitamin E vs. placebo P = 0.0352, *only* vitamin E vs. placebo P = 0.1553; all other smoking-stratified treatment vs. placebo comparisons are P > 0.05.

^†^Mean decline, mL/seconds/year (SD); calculated for men with ≥2 years between first and last pulmonary function tests where date of last test ≤ March 1, 2009; calculated in 418 (placebo), 426 (vitamin E), 416 (selenium) and 417 (vitamin E + selenium) participants.

^††^Model-based estimated mean decline, mL/second/year and 95% confidence interval; main effects models adjusted age, height, race, smoking status, time and tested time x treatment effect (effect of treatment on slope); smoking interaction models adjusted age, height, race, smoking status, and all two way interactions and tested smoking x time x treatment interaction (effect of treatment on slope within smoking group).

^a^P value from mixed-effects linear regression model, with placebo reference group.

^b^Marginal models combine treatment groups. Any selenium model compares participants on any selenium (selenium alone and in combination with E) to placebo. In a separate model, participants on any vitamin E (vitamin E alone and in combination with selenium) are compared to placebo.

In linear mixed-effects regression models adjusted for covariates, the main effect of intervention on rate of decline in lung function was similar to the estimates based on raw data, considering markers of both volume (FEV_1_; Table 3) and flow (FEF_25–75_; Table 4). Thus, in intervention groups the annual rate of decline in FEV_1_ was lower by between 1 and 6 mL/y versus placebo, but effects were not statistically significant (Table 3). Similarly, there were no statistically significant main effects of any intervention on the annual rate of decline in FEF_25–75_ (Table 4). Sensitivity analyses that considered study site or the registration contact at which men started RAS (reflecting the length of time on study intervention) showed similar results.

### Effect modification by cigarette smoking

The hypothesis that smoking modifies the effect of supplementation was pre-specified, and models were extended to estimate intervention effects within categories of cigarette smoking; categories included current, former (quit prior to trial), and never (lifetime never smoker). In the placebo arm, expected differences in rate of decline in FEV_1_ were confirmed such that the annual decline in current smokers was 11 to 16 mL/y steeper compared to former and never smokers (Table 3). Similarly, the annual rate of decline in FEF_25–75_ in current smokers on placebo was more than two-fold that in never smokers (*P* = 0.0189; Table 4).

There was no evidence that smoking modified the effect of intervention on rate of decline in FEV_1_, and all *P* values exceeded the threshold of 0.05. However, for the FEF_25–75_ outcome, compared to placebo the annual rate of decline in FEF_25–75_ was attenuated in current cigarette smokers in the selenium arm (*P* = 0.0219) and in the combined arm (*P* = 0.0236). Further models testing any selenium (selenium alone and selenium + vitamin E, combined) vs. placebo showed that FEF_25–75_ rate of decline was decreased by more than half in current smokers on any selenium compared to current smokers in the placebo group (*P* = 0.0095).

## Discussion

This is the first randomized trial of selenium and/or vitamin E intervention that studies the rate of decline in pulmonary function as the endpoint. This study is important because it contributes new information about whether interventions that presumably affect the antioxidant/oxidant balance in lung tissue can ameliorate or attenuate a functional outcome reflecting lung health. Neither supplementation with selenium nor vitamin E had statistically significant main effects on rate of decline in FEV_1_ or FEF_25–75_. Following our *a priori* hypothesis that effects are stronger in and/or limited to current cigarette smokers, there was evidence for a differential effect of selenium in current smokers for the flow-related endpoint such that smokers supplemented with any selenium, either alone or in combination with vitamin E, had an attenuated rate of decline in FEF_25–75_.

This randomized trial evidence for an effect of selenium on annual rate of decline in lung function in smokers is consistent with prior cross-sectional studies that reported strong positive associations of serum selenium with lung function [14]. An analysis of baseline bloods collected on a subset of SELECT participants [18] found that men were rarely low on serum selenium, where low selenium was defined as ≤ 121.6 ng/mL consistent with prior studies of cancer outcomes [21]. While this suggests the potential-to-benefit from selenium intervention in the overall study may be low, the potential to benefit in smokers is likely to be greater given prior evidence that selenium concentrations are lower in smokers [22], and, indeed, this is supported by our findings.

The pattern of the RAS findings, including the effect of selenium on flow (FEF_25–75_) but not volume parameters and the magnitude of the effect sizes, are similar to the effects of air pollution on lung function reported in the SAPALDIA study. Based on 11 years of follow-up, SAPALDIA reported mean annual rate of decline in FEV_1_ and FEF_25–75_ of 35 mL/y (SD 30) and 71 mL/second/y (SD 65), respectively [23]. In the RAS placebo arm, average annual rates of decline were very similar, although the RAS estimates are more variable given the shorter duration of follow-up and more closely spaced PFTs. SAPALDIA reported that reductions in particulate matter ≤ 10 microns in diameter (PM_10_) were associated with the rate of change in both FEV_1_ and FEF_25–75_, but the strength of the association and the level of statistical significance were greater for the FEF_25–75_ outcome [23], similar to the findings reported herein for supplement effects in RAS. In current smokers the selenium intervention effect size for annual decline in FEV_1_ was similar to the effect size for reducing PM_10_ exposure by 10 μ/m^3^ in SAPALDIA (attenuated FEV_1_ decline by 4 mL/y). Greater effect sizes were seen for FEF_25–75_ in both studies: in SAPALDIA, reducing PM_10_ attenuated FEF_25–75_ decline by 11 mL/second/y, in RAS selenium supplementation attenuated FEF_25–75_ decline by 59 mL/second/y. While FEV_1_ is less variable than FEF_25–75_ in cross-sectional studies [19], longitudinal declines in both endpoints are of interest and FEF_25–75_ findings may be salient in smokers given that changes in flow rates may signal early changes in small airways function [24]. Although such changes may not be predictive at the individual level, they may be informative in the comparison of treatment groups. In addition, a longitudinal endpoint, which leverages repeated measurements per participant, and uses all available spirometry data on each participant (an approach that is consistent with two prior studies of longitudinal change [5,25]), is less affected by variability in comparison to cross-sectional studies.

Although the vitamin E effect sizes were clinically meaningful and consistent in effect direction (attenuated rate of decline) across two pulmonary function measures, with stronger effects of intervention in current smokers, the effects did not meet pre-set criteria for statistical significance. These findings reflect either a true lack of effect of vitamin E on rate of decline in lung function, the possibility that baseline vitamin E levels were high (or at minimum, not deficient) and thus there was limited potential to benefit from supplementation, or the possibility that attenuation in decline might occur only with a longer period of supplementation. While several past observational studies reported that associations of vitamin E were limited to smokers [26], such studies are more likely to be affected by confounding than the randomized trial findings reported herein.

The Respiratory Ancillary Study (RAS) to SELECT used a post-randomization design, and participants were registered to the RAS after active supplementation began. The primary endpoint was rate of decline in lung function; absolute differences in lung function due to the supplements over a fixed period of time were not calculated because first measurements were obtained after the participants started taking their supplements. The design assumes that effects are achieved quickly and are stable over the supplemented period, which is reasonable given the hypothesis of support for antioxidant function provided by the supplements.

This study measured pre-bronchodilator spirometry, but the lack of post-bronchodilator spirometry is not a serious weakness given the primary outcome is rate of decline, which relies on within-person repeated measurements. A recent study shows similar associations of pre- and post-bronchodilator spirometry with mortality [3], which is welcome news given the added participant burden of conducting post-bronchodilator spirometry in healthy population studies.

The strengths of RAS include the enrichment of the study sample with current cigarette smokers, which was part of the *a priori* intent to test effect modification, and the inclusion of a diverse sample (24% African Americans), which supports inference to broader population groups. An additional strength was the extensive infrastructure provided by SELECT, which allowed RAS to be conducted with efficiencies of cost and effort. SELECT infrastructure included an online data collection tool, which allowed incorporation of web-based uploading of spirometry data on a weekly basis, and bi-annual meetings of study personnel, which allowed for optimal training and refresher courses on spirometry methodology.

A few limitations are worth noting. In the post-randomization design, we cannot directly estimate whether the intervention increased FEV_1_ early in the supplementation period in smokers on the active study supplements. This question is important given that prior studies among individuals with COPD show that some clinical treatments increase FEV_1_, but have no effect on FEV_1_ rate of decline [27], and in light of the Lung Health Study, which showed that smoking cessation led to a small but significant initial rebound in FEV_1_, followed by an attenuation in the rate of decline [28]. Our study was conducted in male participants in the SELECT prostate cancer prevention trial, thus whether findings apply to women requires further study. In addition, the design of SELECT did not vary dose and/or formulation, and did not consider whether genetic variation might influence nutrient requirements for optimal health. Finally, the premature termination of supplements meant that final pulmonary function test on some participants was collected well after supplements had been discontinued, and thus analyses were based on fewer participants than originally planned.

## Conclusions

While smoking cessation is the key public health intervention to prevent smoking-related health effects, about 20% of the population continues to smoke [1]. This study investigated the role of nutritional supplementation in lung function decline to identify possible intervention strategies to mitigate lung effects in continuing smokers. This randomized controlled trial found statistically significant protective effects of selenium, specifically 200 μg/d *L*-selenomethionine, on rate of decline in FEF_25–75_ in current cigarette smokers. Supplementation with selenium attenuated the annual rate of decline in FEF_25–75_ in current cigarette smokers, but neither vitamin E nor selenium had effects on rate of decline in FEV_1_. Further studies are needed to understand whether intervention effects are modified by baseline selenium nutriture and/or selenium-related genetic variation [29].

## Additional file

Additional file 1:
**Supplemental Methods.** **Table S1.** The geographic location and number of participants in the Respiratory Ancillary Study (RAS), by SELECT study site, April 2004 through October 2008. **Table S2.** Spirometry Testing: Description of Pulmonary Function Test Measurements and Quality Control Indicators in the Respiratory Ancillary Study to SELECT, April 2004 to October 2008. **Table S3.** Linear mixed-effects regression model for main effects of treatment on FEV_1_ and FEF_25–75_ for model testing each treatment arm vs. placebo. **Table S4.** Linear mixed-effects regression model for effects of treatment on FEV_1_ and FEF_25–75_ including test of smoking by treatment interaction. **Figure S1.** Distribution of Annual Change in Forced Expiratory Volume in the First Second (FEV_1_)^1^ for Participants in the Respiratory Ancillary Study (RAS) to SELECT.

## Abbreviations

FEV_1_: Forced expiratory volume in the first second
FEF_25–75_: Forced expiratory flow
COPD: Chronic obstructive pulmonary disease
SELECT: Selenium and Vitamin E Cancer Prevention Trial
RAS: The Respiratory Ancillary Study to SELECT
