# Supplementary material for: A randomized controlled trial of vitamin E and selenium on rate of decline in lung function
Source: Respir Res. 2015 Mar 11;16(1):35. doi: 10.1186/s12931-015-0195-5 (PMC4404242; doi:10.1186/s12931-015-0195-5)
Supplement: Additional file 1: — Supplemental Methods. Table S1. The geographic location and number of participants in the Respiratory Ancillary Study (RAS), by SELECT study site, April 2004 through October 2008. Table S2. Spirometry Testing: Description of Pulmonary Function Test Measurements and Quality Control Indicators in the Respiratory Ancillary Study to SELECT, April 2004 to October 2008. Table S3. Linear mixed-effects regression model for main effects of treatment on FEV1 and FEF25–75 for model testing each treatment arm vs. placebo. Table S4. Linear mixed-effects regression model for effects of treatment on FEV1 and FEF25–75 including test of smoking by treatment interaction. Figure S1. Distribution of Annual Change in Forced Expiratory Volume in the First Second (FEV1)1 for Participants in the Respiratory Ancillary Study (RAS) to SELECT. [file 12931_2015_195_MOESM1_ESM.docx]

A Randomized Controlled Trial of
Vitamin E and Selenium on Rate of Decline in Lung Function

Patricia A Cassano^1,2^, Kristin A Guertin^1^, Alan R Kristal^3,4^, Kathryn A Ritchie^1^, Monica L Bertoia^1^, Kathryn B Arnold^5^, John J Crowley^5^, JoAnn Hartline^5^, Phyllis J Goodman^5^, Catherine M Tangen^3^, Lori M Minasian^6^, Scott M Lippman*^7^,
Eric Klein*^8^

^1^ Division of Nutritional Sciences, Cornell University, Ithaca, NY, USA

^2^Department of Public Health, Division of Biostatistics and Epidemiology, Weill Cornell Medical College, NY, NY, USA

^3^Department of Epidemiology, University of Washington, Seattle, WA, USA

^4^Fred Hutchinson Cancer Research Center, Seattle, WA, USA

^5^ SWOG Statistical Center, Seattle, WA, USA

^6^Division of Cancer Prevention, National Cancer Institute, Bethesda, MD, USA

^7^University of California San Diego, Moores Cancer Center, La Jolla, CA, USA

^8^Cleveland Clinic, Cleveland, OH, USA

Corresponding and requests for reprints should be addressed to Dr. P.A. Cassano, 209 Savage Hall, Cornell University, Ithaca NY 14853 USA; (607) 255-7551; email [pac6@cornell.edu](mailto:pac6@cornell.edu)

*These authors contributed equally to this work

**Supplemental Methods**

Planning for the Study Sample Size

Power calculations were performed in planning the study to estimate the number of participants necessary to detect the hypothesized effects. The power calculations were based on the difference in the change in FEV_1_ between baseline and follow-up between treatment groups, often termed the “intervention effect”. We hypothesized an intervention effect over 3 years of 85mL, 128mL and 43mL for selenium alone, the combination of selenium and vitamin E, and vitamin E alone, all compared to placebo, respectively. With a sample size of 3,000 participants (750 in each arm) we estimated the power would be 90, >99 and 30%, for selenium alone, the combination of selenium and vitamin E, and vitamin E alone, respectively.

Pulmonary Function Tests (PFTs)

SELECT clinical research nurses were trained in spirometry by the RAS staff; bi-annual refresher sessions were required, all lung function tests were reviewed centrally by RAS staff, and weekly feedback was provided to the SELECT site team to constantly improve data collection. Participants with a minimum of 24 months between repeated PFTs were included in longitudinal models; follow-up PFTs through four months after supplementation ended were included, which is a conservative bias.

Study Supplements

The SELECT Pharmacy Coordinating Center assured supplement quality. Further details about supplement quality control and quality assurance are available elsewhere[[1](#_ENREF_1)].

Statistical analysis

A linear mixed-effects model was used to model the longitudinal trajectory of FEV_1_ (i.e. baseline FEV_1_ and its rate of change over time). A continuous time (slope) variable was included in the model to quantify the time (in years) elapsed between each FEV_1_ measurement and the study baseline and the coefficient for time conveyed the rate of change in FEV_1_ (mL/year). To allow for variation in baseline FEV_1_ and its rate of change across participants, the intercept and time variable were specified as both fixed and random effects. The model allows flexibility in handling unbalanced longitudinal data, and allows inclusion of participants with only one FEV_1_ measurement in addition to those with more than one measurement.

The model was adjusted for baseline age, baseline height and baseline smoking pack-years, smoking status during study follow-up, and the product term of smoking status x time. Smoking status during study follow-up was defined as a four-level categorical variable as follows, based on data collected biannually: never smokers at all time points (referent group), persistent smokers (current smokers at all time points), former smokers at all time points, and intermittent smokers (inconsistent smoker status across time points). Models further adjusted for study site, and for entry time into the study had little or no effect on treatment effects, thus simpler models are presented herein. The above main effects estimated how these covariates affected baseline FEV_1_ and the product term of smoking status x time estimated the effect of the smoking on the rate of change in FEV_1_ (for each of 3 smoking groups in comparison to never smokers). Given the complexity in the model, the rate of decline in lung function is computed for each treatment group, based on model coefficients. In main effect models, the time variable conveys the rate of decline in the placebo group, and the addition of the time coefficient and the time-by-treatment coefficient conveys the rate of change in the treatment group (for example, using data in Supplemental Table 3, rate of decline in FEV_1_ in the placebo group was -39.20mL/y, rate of decline in the selenium group was -39.20 + 1.55, or –37.65mL/y).

Using the above model, we evaluated the presence and magnitude of covariance between the random intercept and time effects; given no meaningful covariance was present, the two random effects were specified as independent for model parsimony. Thus, the optimal variance-covariance structure was variance components (identified using AIC criteria). Subsequently, residual diagnosis was pursued based on the same preliminary model to exclude FEV_1_ measurements detected as outliers (i.e. |standardized residual| > 4), and plots of studentized residuals versus predicted values identified no difference in residuals by treatment arm.

**Supplemental Tables and Figures**

**Supplemental Table 1.** The geographic location and number of participants in the Respiratory Ancillary Study (RAS), by SELECT study site, April 2004 through October 2008.

| **Study Site Name** | **Location** | **Number** | **(%)** |
| --- | --- | --- | --- |
| Altamira Family Medicine | San Juan , Puerto Rico | 35 | (1.2) |
| Le Centre de Recherche | Quebec, Canada | 155 | (5.3) |
| Harbor, University of California Los Angeles | Torrance, California | 292 | (10) |
| London Health Sciences Center/Regional Cancer Center | Ontario, Canada | 189 | (6.5) |
| MD Anderson | Houston, Texas | 149 | (5.1) |
| Rush University Medical Center | Chicago, Illinois | 140 | (4.8) |
| SUNY Stonybrook | E. Setauket, New York | 197 | (6.8) |
| Swedish Medical Center | Seattle, Washington | 327 | (11.2) |
| University of California, San Diego, Chula Vista* | La Jolla, California | 190 | (6.5) |
| Upstate Carolina | Spartanburg, South Carolina | 201 | (6.9) |
| Veterans Administration (VA) Medical Center Jesse Brown | Chicago, Illinois | 494 | (16.9) |
| VA Medical Center, Kansas City | Kansas City, Missouri | 77 | (2.6) |
| VA Medical Center, Minneapolis | Minneapolis, Minnesota | 217 | (7.4) |
| VA Medical Center, Puget Sound | Seattle, Washington | 54 | (1.8) |
| VA Medical Center, Washington DC | Washington, District of Columbia | 72 | (2.5) |
| Wichita CCOP | Wichita, Kansas | 131 | (4.5) |

*Chula Vista and University of California, San Diego were originally two separate sites, and were combined for administrative efficiencies during SELECT follow-up

**Supplemental Table 2.** Spirometry Testing: Description of Pulmonary Function Test Measurements and Quality Control Indicators in the Respiratory Ancillary Study to SELECT, April 2004 to October 2008*.

|  | Placebo  (n= 726) | Vitamin E  (n= 726) | Selenium  (n=759 ) | Vitamin E and Selenium  (n= 709) |
| --- | --- | --- | --- | --- |
| No. of tests completed^a^ | 2,442 | 2,410 | 2,532 | 2,327 |
| **FEV_1_** |  |  |  |  |
| Quality Score, all tests^a^ | 3.5 (0.9) | 3.5 (0.8) | 3.5 (0.9) | 3.5 (0.8) |
| Quality Score, acceptable tests^b^ | 3.7 (0.4) | 3.7 (0.5) | 3.7 (0.4) | 3.7 (0.4) |
| Quality Score, model decline^c^ | 3.7 (0.4) | 3.7 (0.4) | 3.7 (0.4) | 3.7 (0.4) |
| **FVC** |  |  |  |  |
| Quality Score, all tests^a^ | 3.3 (1.1) | 3.3 (1.0) | 3.2 (1.1) | 3.3 (1.0) |
| Quality Score, acceptable tests^d^ | 3.7 (0.5) | 3.7 (0.5) | 3.7 (0.5) | 3.7 (0.5) |

^a^all tests completed following ATS standardization guidelines, shown here regardless of quality score or acceptable start and end of test; quality score 0=unacceptable start or end of test; 1=repeated trials >250mL ; 2=repeated trials within 250mL ; 3=repeated trials within 150mL; 4=repeated trials within 75 mL;

^b^limit tests to acceptable start of test (no hesitation), and quality score > 3; number of tests 2172, 2147, 2218, and 2101 for placebo, E, selenium, E + selenium, respectively

^c^ limit tests to time period of supplementation (< March 1 2009), acceptable start of test (no hesitation), quality score > 3 and repeated measures for models of decline; number of tests 1594, 1606, 1596, and 1565 for placebo, E, selenium, E + selenium, respectively

^d^ limit tests to acceptable end of test, and quality score > 3; number of tests 2067, 2072, 2142, and 1993 for placebo, E, selenium, E + selenium, respectively

*FEF_25-75_ is based on QC for both FEV_1_ and FVC, so only tests that have acceptable start of test, acceptable end of test and QC > 3 for both FEV_1_ and FVC contribute to models on FEF_25-75_

**Supplemental Table 3.** Linear mixed-effects regression model for main effects of treatment on FEV_1_ and FEF_25-75_ for model testing each treatment arm vs. placebo.

| **Model Variables** | **Beta** | **SE** | **P Value** |
| --- | --- | --- | --- |
| **A. Outcome: FEV_1_ decline** |  |  |  |
| Time, elapsed years since baseline | -39.20 | 3.44 | <0.001 |
| Age at SELECT Baseline | -40.42 | 1.62 | <0.0001 |
| Height at first PFT | 31.36 | 1.43 | <0.0001 |
| Race (African American) | -501.22 | 25.03 | <0.0001 |
| Smoking Status: |  |  |  |
| Intermittent/Persistent Smokers | -364.51 | 29.97 | <0.0001 |
| Former Smokers | -128.22 | 22.61 | <0.0001 |
| Never Smokers | Reference | - | - |
| Treatment: |  |  |  |
| Vitamin E | -58.84 | 31.18 | 0.0592 |
| Selenium | -45.79 | 30.99 | 0.1397 |
| Vitamin E +Selenium | -25.11 | 31.40 | 0.4239 |
| Placebo/Placebo | Reference | - | - |
| Time * Treatment: |  |  |  |
| Time * Vitamin E | 6.36 | 4.82 | 0.1866 |
| Time * Selenium | 1.55 | 4.87 | 0.7502 |
| Time * Vit. E + Selenium | 4.92 | 4.88 | 0.3133 |
| Time * Placebo | Reference | - | - |
|  |  |  |  |
| **B. Outcome: FEF_25-75_ decline** |  |  |  |
| Time, elapsed years since baseline | -60.59 | 8.87 | <0.0001 |
| Age at SELECT Baseline | -51.27 | 2.63 | <0.0001 |
| Height at first PFT | 18.61 | 2.32 | <0.0001 |
| Race (African American) | -491.12 | 40.72 | <0.0001 |
| Smoking Status: |  |  |  |
| Intermittent/Persistent Smokers | -738.62 | 48.33 | <0.0001 |
| Former Smokers | -228.10 | 36.60 | <0.0001 |
| Never Smokers | Reference | - | - |
| Treatment: |  |  |  |
| Vitamin E | -62.65 | 59.47 | 0.2922 |
| Selenium | 29.89 | 59.31 | 0.6143 |
| Vitamin E +Selenium | -25.46 | 60.26 | 0.6727 |
| Placebo | Reference | - | - |
| Time * Treatment: |  |  |  |
| Time * Vitamin E | 13.53 | 12.37 | 0.2739 |
| Time * Selenium | -5.47 | 12.51 | 0.6620 |
| Time * Vit. E + Selenium | 13.82 | 12.57 | 0.2719 |
| Time * Placebo | Reference | - | - |

**Supplemental Table 4.** Linear mixed-effects regression model for effects of treatment on FEV_1_ and
FEF_25-75_ including test of smoking by treatment interaction.

| **Model Variables** | **Beta** | **SE** | **P Value** |
| --- | --- | --- | --- |
| **A. Outcome: FEV_1_ decline** |  |  |  |
| Time, years since baseline | -40.43 | 5.64 | <0.0001 |
| Age at SELECT Baseline | -40.34 | 1.62 | <0.0001 |
| Height at first PFT | 31.32 | 1.43 | <0.0001 |
| Race (African American) | -500.60 | 25.08 | <0.0001 |
| Smoking Status (never=reference) |  |  | Type III =<0.0001 |
| Intermittent/Persistent Smokers | -345.54 | 63.46 | <0.0001 |
| Former Smokers | -164.91 | 49.31 | 0.0008 |
| Never Smokers | Reference | - | - |
| Treatment (placebo=reference) |  |  | Type III= 0.2788 |
| Vitamin E | -77.83 | 52.55 | 0.1387 |
| Selenium | -66.39 | 51.03 | 0.1933 |
| Vitamin E + Selenium | -46.05 | 53.05 | 0.3855 |
| Placebo | Reference | - | - |
| Time * Treatment |  |  | Type III= 0.2763 |
| Time * Vitamin E | 9.36 | 8.08 | 0.2469 |
| Time * Selenium | 3.22 | 8.04 | 0.6886 |
| Time * Vit.E + Selenium | 8.54 | 8.32 | 0.3045 |
| Time *Smoking Status |  |  | Type III =0.4406 |
| Current | -10.53 | 10.05 | 0.2951 |
| Former | 5.92 | 7.61 | 0.4369 |
| Smoking Status * Treatment |  |  | Type III = 0.9511 |
| Current* Vitamin E | 10.00 | 88.63 | 0.9101 |
| Former* Vitamin E | 38.15 | 69.96 | 0.5856 |
| Current* Selenium | 6.85 | 88.66 | 0.9384 |
| Former* Selenium | 43.24 | 68.95 | 0.5306 |
| Current* Vitamin E+Selenium | -33.97 | 89.00 | 0.7027 |
| Former* Vitamin E+Selenium | 59.22 | 70.60 | 0.4016 |
| Time * smoking Status * Treatment |  |  |  |
| Time*Current*Vitamin E | 12.80 | 13.85 | 0.3552 |
| Time*Former*Vitamin E | -10.73 | 10.78 | 0.3199 |
| Time*Current*Selenium | 9.46 | 13.98 | 0.4985 |
| Time*Former*Selenium | -6.40 | 10.86 | 0.5555 |
| Time*Current*Vitamin E + Selenium | -1.96 | 14.03 | 0.8887 |
| Time*Former*Vitamin E + Selenium | -5.61 | 10.98 | 0.6095 |
|  |  |  |  |
| **B. Outcome: FEF_25-75_ decline** |  |  |  |
| Time, Years Since Baseline | -43.89 | 14.89 | 0.0032 |
| Age at SELECT Baseline | -51.22 | 2.63 | <0.0001 |
| Height at first PFT | 18.44 | 2.32 | <0.0001 |
| Race (African American) | -490.84 | 40.76 | <0.0001 |
| Smoking Status (never=reference) |  |  | Type III |
| Intermittent/Persistent Smokers | -597.38 | 122.70 | <0.0001 |
| Former Smokers | -277.17 | 94.40 | 0.0033 |
| Never Smokers | Reference | - | - |
| Treatment (placebo=reference) |  |  |  |
| Vitamin E | -81.00 | 100.78 | 0.4216 |
| Selenium | 35.27 | 98.57 | 0.7205 |
| Vitamin E + Selenium | 10.70 | 102.16 | 0.9166 |
| Placebo | Reference | - | - |
| Time * Treatment |  |  |  |
| Time * Vitamin E | -3.08 | 21.13 | 0.8841 |
| Time * Selenium | -22.65 | 20.94 | 0.2795 |
| Time * Vitamin E + Selenium | -27.00 | 21.74 | 0.2144 |
| Time*Smoking Status |  |  |  |
| Current | -61.69 | 26.25 | 0.0189 |
| Former | -12.85 | 19.65 | 0.5132 |
| Smoking Status * Treatment |  |  | Type III |
| Current, Vitamin E | -61.74 | 169.15 | 0.7151 |
| Former, Vitamin E | 56.81 | 133.66 | 0.6708 |
| Current, Selenium | -263.58 | 169.68 | 0.1204 |
| Former, Selenium | 91.97 | 132.23 | 0.4868 |
| Current, Vitamin E+Selenium | -140.91 | 172.54 | 0.4141 |
| Former, Vitamin E+Selenium | -21.31 | 135.22 | 0.8748 |
| Time * Smoking Status * Treatment |  |  |  |
| Time*Current*Vitamin E | 50.78 | 35.60 | 0.1539 |
| Time*Former*Vitamin E | 17.58 | 27.78 | 0.5269 |
| Time*Current*Selenium | 83.18 | 36.27 | 0.0219 |
| Time*Former*Selenium | 4.75 | 27.83 | 0.8644 |
| Time*Current*Vitamin E + Selenium | 83.05 | 36.66 | 0.0236 |
| Time*Former*Vitamin E + Selenium | 53.66 | 28.28 | 0.0579 |


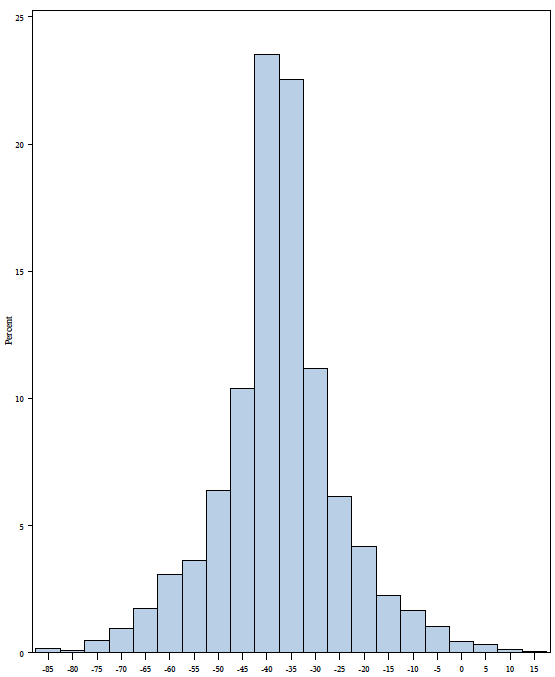


**Supplemental Figure 1** Distribution of Annual Change in Forced Expiratory Volume in the First Second (FEV_1_)^1^ for Participants in the Respiratory Ancillary Study (RAS) to SELECT

^1^Estimates from linear mixed-effects model including treatment, time and treatment by time, mean slope is -37.5 (SD 12.5)

References:

1. Lippman SM, Klein EA, Goodman PJ, Lucia MS, Thompson IM, Ford LG, Parnes HL, Minasian LM, Gaziano JM, Hartline JA, et al: **Effect of selenium and vitamin E on risk of prostate cancer and other cancers: the Selenium and Vitamin E Cancer Prevention Trial (SELECT).** *JAMA* 2009, **301:**39-51.
